# Pathways related to the degeneration of health behavior in stroke patients: a qualitative comparative analysis

**DOI:** 10.3389/fpubh.2026.1769132

**Published:** 2026-04-24

**Authors:** Lei Chen, Yi Zhang, Jia-Xuan Li, Wei-Ying Zhong, Xi Pan, Lan Xu

**Affiliations:** Departments of Neurology, The First Affiliated Hospital of Soochow University, Suzhou, Jiangsu, China

**Keywords:** csQCA, health behavior, qualitative comparative analysis, self-regulation, stroke

## Abstract

Stroke is characterized by a high recurrence rate, which can cause significant physical and psychological harm to patients. The implementation of specific health behaviors represents an effective measure to reduce the recurrence rate; however, the current status of specific health behavior implementation in stroke patients is not optimal. The evolution of certain health behaviors is a complex process. However, previous studies have predominantly focused on isolated variables and, as a direct consequence, cannot fully explain the underlying mechanisms involved, thus hindering the development of effective interventions. To solve this problem, we adopted the crisp-set qualitative comparative analysis (csQCA) method from a configurational perspective to analyze the health behavior cases of 210 stroke patients. We investigated the complex influences between these variables and the outcome variable by performing univariate necessity analysis and path configuration analysis. The analysis revealed no single necessary condition. We identified seven sufficient pathways, categorized as endogenous (dominated by self-control and self-regulation deficits) or exogenous (driven by external misinformation). Robustness tests confirmed a stable core configuration (low self-control × insufficient motivation × self-control weakening) within the endogenous pathways. This underscores that degradation stems from specific combinations of conditions, not isolated factors. Our findings suggest that we should focus on identifying and addressing multiple conditional variables from a holistic perspective, with particular attention to a patient’s self-control ability and the accuracy of external information sources. Future intervention programs may be developed by considering the specific combination of variables identified in each pathway. Our findings advocate for a shift from single-factor to configurational, multi-level interventions. These should simultaneously target the stable psychological core and mitigate diverse contextual risks. This study provides a hierarchical framework for understanding health behavior degradation, offering a basis for personalized intervention strategies.

## 1 Introduction

In a previous report, the World Health Organization (WHO) ranked stroke as the second leading cause of death worldwide, accounting for approximately 11% of all deaths worldwide (1). Stroke places a huge burden on both individuals and society; previous studies have estimated that the total direct medical costs associated with stroke will increase from $36.7 billion to $94.3 billion between 2015 and 2035 (2). The risk of stroke recurrence is high, with a 5-year recurrence rate of 16.0–20.1% (3). Furthermore, the recurrence of stroke is associated with more severe neurological damage than the first episode; it is more difficult to treat, and carries a higher risk of death (4). The danger of stroke recurrence cannot be ignored. Over recent years, the development of various interventions has reduced the morbidity and mortality rates associated with stroke, but has not led to a significant improvement in recurrence rate (5).

The adoption of healthy behaviors is an important aspect of secondary stroke prevention. According to the 2023 report published by the American Heart Association, healthy behaviors for stroke patients include abstaining from smoking and alcohol intake, increasing exercise, eating a healthy diet, sleeping for appropriate durations, and taking medications as prescribed by a doctor (6). The efficient adoption of healthy behaviors can exert significant effects on recovery and the prevention of recurrence in stroke patients (7). However, the current status of health behaviors in stroke patients is not optimal. A previous study followed the health behaviors of stroke patients for 6 months after discharge from hospital and found that health behaviors improved over the first 3 months post-discharge but then declined significantly (8). Other studies showed that over time, many stroke patients have a tendency to down-grade their health behaviors post-discharge (9), including physical inactivity (10), poor diets (11), smoking (12), and reduced drug compliance (13). The degradation of health behavior can affect functional recovery (14), significantly increase the risk of stroke recurrence (14, 15), and may also increase the risk of other cardiovascular diseases (12). Therefore, maintaining specific health behaviors is a key and urgent challenge in the prevention of stroke recurrence. It is noteworthy that different health behaviors (e.g., smoking cessation, exercise, dietary control) may involve distinct mechanisms for initiation and maintenance. However, stroke secondary prevention in clinical practice constitutes an integrated management regimen, requiring patients to address multiple behavioral changes simultaneously (16). This study does not aim to delineate the unique determinants of individual behaviors. Instead, it seeks to identify the common configurations of conditions that lead to a generalized deterioration of patients’ established health behavior regimens. In other words, we focus on the “failure of behavioral maintenance” rather than “behavioral initiation,” aiming to pinpoint the common vulnerability pathways that may trigger synchronous decline across multiple behaviors.

The development of healthy behaviors is a long-term and complex process that does not follow a strict format. The key to maintaining a high level of healthy behavior is to prevent the degradation of healthy behavior. The factors that control the degradation of health behavior are intricate and fall into two main categories: disease factors and non-disease factors. Disease factors predominantly relate to a range of physical discomforts associated with stroke, such as cognitive dysfunction (6), walking disorders (17) and communication disorders (18). These factors can reduce a patient’s willingness to perform health behaviors (18) and increase the difficulty of performing health behaviors (6, 17), thus resulting in the degradation of health behavior. Non-disease factors include the patient’s own factors, along with social and family factors. In a previous study, researchers detected a positive correlation between a patient’s negative perception of disease and a low health behavior score (14). The more severe a patient’s negative perception of the illness, the more likely the patient is to believe that they cannot change their illness, thus leading to burnout and an inability to adhere to healthy behaviors. Studies have also shown that there is a significant positive correlation between health belief, health literacy and health behavior. The better the health belief, the higher the level of health literacy and the lower the possibility of health behavior degradation (19, 20). Moreover, the psychological status of stroke patients is predictive of their lifestyle and behavior (21); a poorer psychological status leads to reduced social participation and exercise, and an increased risk of health behavior degradation (22). Social and family support is also one of the most important factors influencing the degradation of health behavior. A lack of family and other types of support can impede the implementation of healthy behaviors and the maintenance of a healthy lifestyle (23, 24). Previous research identified and demonstrated a number of factors affecting the health behavior of stroke patients. However, most of these studies have only examined one-sided correlations between different factors and health behaviors, while a few have explored potential mediatory effects. Consequently, our existing knowledge remains limited. The development and change of health behavior degradation result from the combination of many factors. The specific process and causality of health behavior degradation cannot be fully explained by investigating single factors in isolation. Identifying the specific pathways leading to health behavior degradation in stroke patients is a prerequisite for effective intervention. Interventions based solely on single correlation or mediation analyses cannot effectively improve the levels of health behavior. Therefore, in the present study, we utilized qualitative comparative analysis (QCA), a case-oriented research method that bridges qualitative and quantitative analysis (25). This method employed a configurational perspective that is commonly used to understand causal complexity in organizational outcomes (26), which posits that multiple influences are interdependent and can be aligned in different ways to achieve the common purpose of influencing organizational outcomes (26). QCA adopts a set-theoretic approach that conceptualizes causal conditions and outcome variables as sets, revealing complex causal relationships by analyzing the sufficiency and necessity of conditions or their combinations (27). QCA can effectively address causal complexity in health systems (28). Here, we applied QCA to the study of health behaviors in stroke patients for the first time, offering new perspectives for future research on complex health management issues. Our analysis identifies specific pathways underlying the degradation of health behavior in stroke patients and provide a theoretical basis for the development of effective strategies to prevent the recurrence of stroke.

## 2 Materials and methods

### 2.1 Methodology

**Figure 1:**
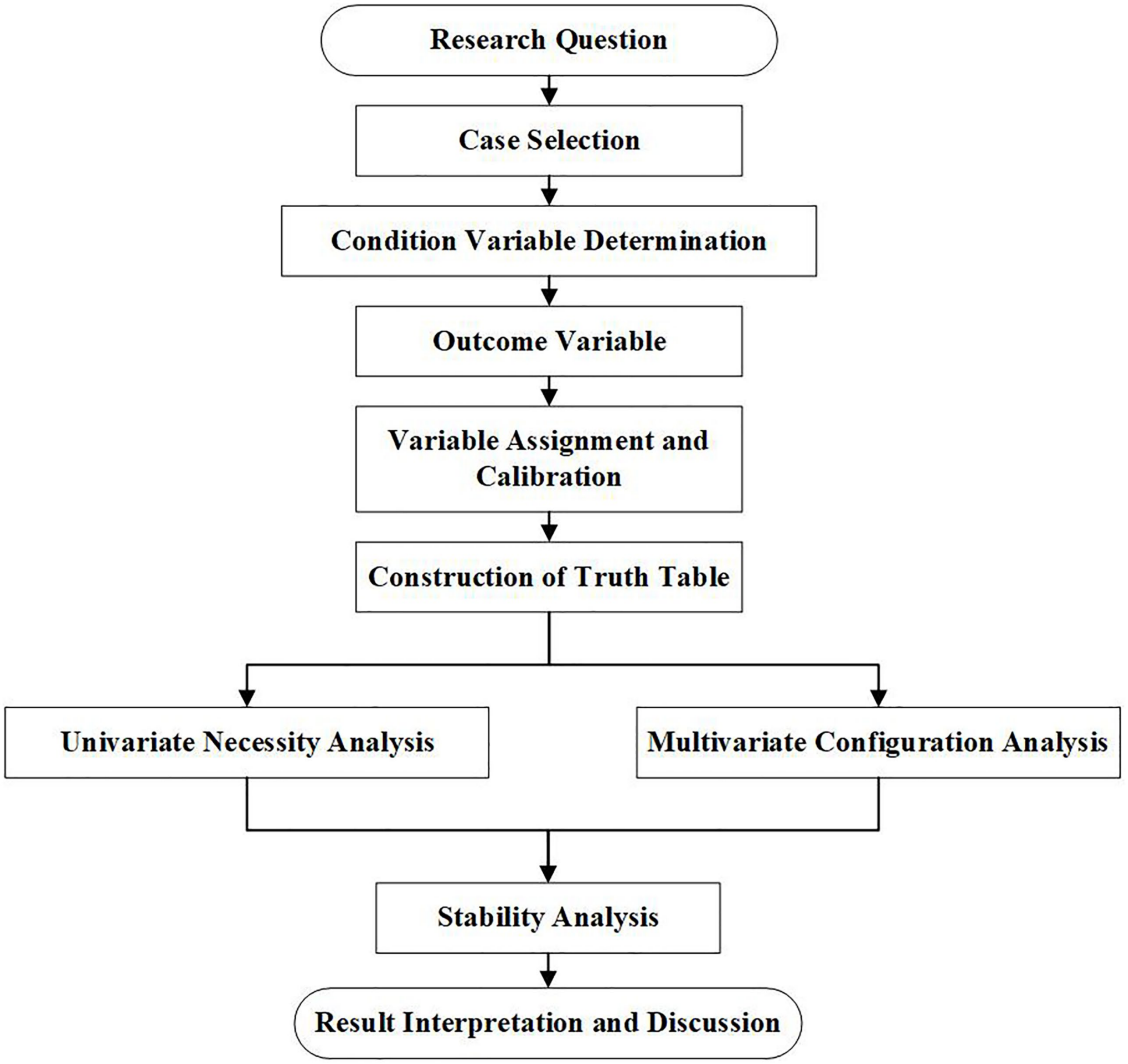
Flowchart of the csQCA analysis process.

QCA represents a third option that falls between qualitative and quantitative research. The essence of QCA is a Boolean algebra algorithm that analyzes the internal logic between factors from a holistic and systematic perspective. This method is highly suitable for dealing with multiple interactions between multiple variables, thus breaking the limitations of linear relationships and investigating multiple driving mechanisms of interactions between key factors from the perspective of multi-factor grouping (29). During its initial phase, this research method was predominantly used in social science disciplines such as sociology and political science to explain the causes of social phenomena in a holistic and combinatorial manner. Our main reason for selecting the QCA approach to investigate the pathways underlying the degradation of health behavior in stroke patients in this study is that previous studies of health behavior in stroke patients, whether qualitative or quantitative, generally investigated the relationship between individual factors and health behavior. Furthermore, the conclusions of these previous studies were limited to a static perspective, pursuing only the optimal single causal model, with elements existing independently of each other in a separate form, thus preventing a critical understanding of how these factors interact. In contrast, the QCA approach can analyze the multiple mechanisms that drive the degradation of health behavior in stroke patients from a group perspective, which can be demonstrated from three aspects. First, QCA focuses on multiple combinations of conditions for a specific phenomenon and is used to address the situation in which a phenomenon is not due to a single cause; where a change in a cause does not necessarily lead to a change in the corresponding outcome; and where the same phenomenon may be caused by different combinations of conditions (26). The degradation of health behaviors in stroke patients is a complex and multifactorial process that features many cross-interactions. By using the QCA method, we can investigate the various combinations of conditions that can lead to the degradation of health behaviors in stroke patients at the group level. Furthermore, we can investigate the mechanisms that lead to the degeneration of health behavior in patients from a more comprehensive and holistic perspective. Second, the QCA method is based on the principle of equifinality (25) and can identify the multiple pathways responsible for the deterioration of health behavior in stroke patients to provide appropriate solutions for healthcare professionals to help patients improve and maintain their health behaviors. Third, the QCA approach is applicable to small and medium-sized case studies (25), and has been developed for large samples (26). This method is less limited in terms of sample size, and the analysis of its results does not depend on the size of the sample; rather, it relies on whether the sample size covers representative individuals. The sample population of this study was stroke patients discharged from a tertiary hospital between 2022 and 2023, and the sample size of the study was appropriate to meet the requirements of the QCA method. A standard flowchart illustrating the key steps of the csQCA analysis process adopted in this study is provided in Figure 1.

### 2.2 Case selection

Initially, we employed a longitudinal study design to acquire suitable cases for QCA research. We established four specific time points for investigation: discharge (Time 1, T1), 3 months post-discharge (Time 2, T2), 6 months post-discharge (Time 3, T3), and 12 months post-discharge (Time 4, T4). The health behavior levels of stroke patients were assessed at each designated time point. The participants were stroke patients admitted to a Grade III hospital in Suzhou City between July 2022 and June 2023. The inclusion criteria for participants were as follows: (i) individuals diagnosed with stroke (either ischemic, hemorrhagic, or the conjunction of both); (ii) normal understanding and communication skills; (iii) interested in the study and willing to participate in the study. The exclusion criteria were as follows: (i) the presence of other severe underlying conditions or complications; (ii) major psychiatric disorders; and (iii) hospitalization due to illnesses that were not related to stroke. In order to ensure the stability of parameter estimation for the latent variable model, the sample size needed to be ≥200 (30). Considering a 15% loss of follow-up rate, a sample size of at least 230 people needed to be included. Ultimately, we included 340 subjects, and finally completed follow-up in 276 of these patients; the loss to follow-up was 18.8%.

The questionnaire consisted of two parts. The first part collected sociodemographic and clinical data. In the second part, we used the stroke patient health behavior scale compiled by Rong et al. (31) to evaluate the level of health behavior in stroke patients. The scale was designed by Rong et al., which consists of 24 items with five dimensions, including basic health behavior, health care behavior, early warning behavior, avoiding harmful environmental behavior, and abstaining from bad habits. Each item is scored on a five-point Likert scale, with potential total scores ranging between 24 and 120 points. The scale has demonstrated reliability among patients with stroke (Cronbach’s *α* = 0.807), and the Cronbach’s α was found to be 0.864 in our sample (31).

**Figure 2:**
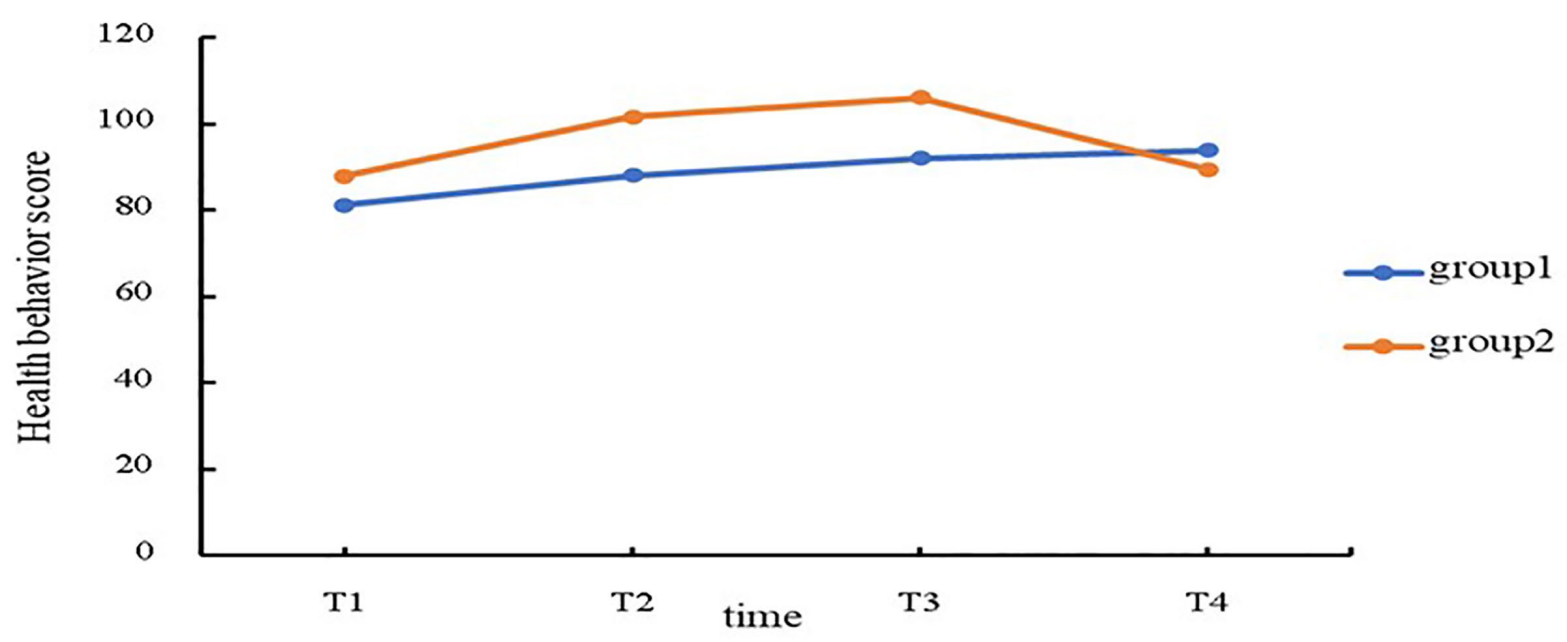
Longitudinal trajectories of health behavior scores across four time points (T1-T4) for the identified subgroups.

In order to investigate the combination of causal pathways underlying the degradation of health behavior in stroke patients, it is necessary to select appropriate specific stroke cases, including both positive cases (well-maintained health behaviors) and negative cases (poor health behaviors). We utilized data from the longitudinal survey to construct a latent variable growth mixture model (GMM) to delineate categories of the longitudinal health behavior trajectory in stroke patients 1 year after discharge. The model was estimated using the maximum likelihood robust estimator (MLR). The indices used to fit the model included Akaike Information Criterion (AIC), Bayesian Information Criterion (BIC), α Bayesian Information Criterion (αBIC), Entropy, Logarithm likelihood ratio statistic (LMR), and the bootstrap-based likelihood ratio test (BLRT). Smaller values for the AIC, BIC and αBIC indicate a better model fit. Entropy ranges from 0 to 1; higher entropy values signify greater classification accuracy. Significance (P) values corresponding to LMR and BLRT indicate that the k-profile model outperforms the K-1 profile model (30). Results are presented in Table 1; based on these indices, we selected two categories as the optimal model division. The model trajectory is depicted in Figure 2. The two categories were defined as patient groups in which health behavior was rising or declining. The outcome of the case was determined by the patient’s group. Patients in the increasing health behavior group (178 patients) were selected as an alternative sample for studying positive cases, and patients in the declining health behavior group (98 patients) were selected as an alternative sample for studying negative cases. The questionnaires were matched to specific patients based on their personal information, home address, and contact information, and we communicated with the alternative stroke patients by utilizing contact information given in the questionnaires and scheduled telephone interviews. Ultimately, a total of 210 patients participated in questionnaire surveys. A total of 126 positive cases and 84 negative cases completed the surveys. QCA analysis combines the complementary strengths of qualitative and quantitative research, and is not demanding in terms of the number of cases required; initially, this method was used in studies featuring small to medium case numbers (10–50) (25). However, in recent years, this methodology has been deemed suitable for studies with larger samples exceeding 100 cases (26).

**Table 1:** Fitting results for the trajectory of health behavior in stroke patients.

Class	AIC	BIC	αBIC	LMR(p)	BLRT(p)	Entropy	Group size
Class 1	8777.943	8825.008	8783.788				
Class 2	8765.798	8827.345	8773.441	0.0217	0	0.688	178/98
Class 3	8769.635	8845.664	8779.076	0.6665	1	0.551	73/105/98

### 2.3 Research modeling

**Figure 3:**
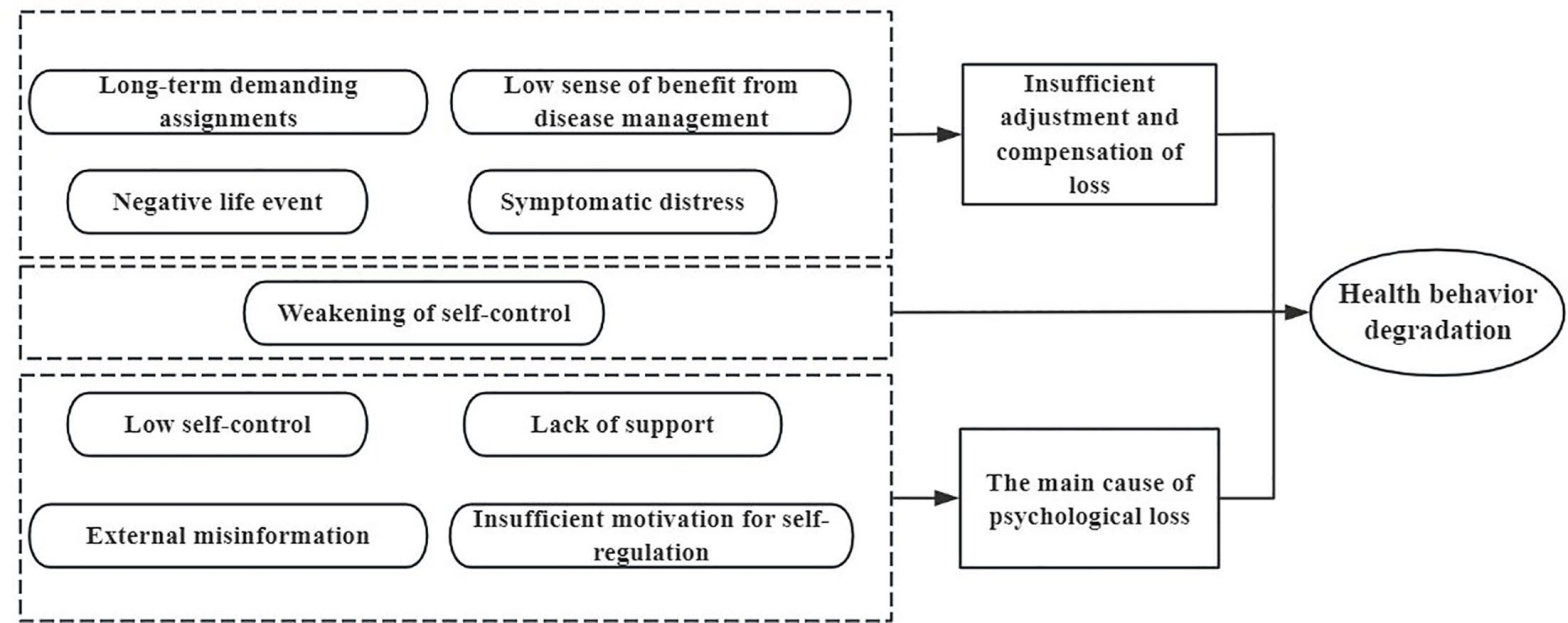
Conceptual framework of the core and contextual conditions leading to health behavior degradation.

The research model for this paper was derived from a previous qualitative study conducted by our group which summarized the causes of health behavior degradation in stroke patients in accordance with the ego depletion theory; this included three themes and nine causes (32). The three main themes relating to the causes of health behavior degradation in stroke patients under the ego depletion theory were the depletion of psychological resources, insufficient regulation and the compensation of depletion, and weakened levels of self-control (Figure 3). The relationship between the three qualitative themes and the nine specific conditions is visualized in Figure 3. Based on our prior qualitative study, the dashed boxes categorize each condition under the theme to which it was originally attributed. This figure serves solely as a conceptual map to illustrate the thematic provenance of each variable. The subsequent QCA examines how these variables configure into distinct, sufficient pathways leading to the outcome.

### 2.4 Variable definition, measurement, and calibration

#### 2.4.1 Variable selection and definitions

Based on our prior qualitative study (32), we identified nine core barrier factors. These are specified below.

1. Long-term high-demand tasks refer to the complex self-management requirements following stroke. Research indicates that patients with chronic conditions, including stroke, face a significantly higher risk of medication non-adherence when they experience a high treatment burden (33). Furthermore, empirical studies have shown that a greater treatment burden is linked to poorer self-management among stroke survivors (34).
2. Symptomatic distress denotes the physical discomfort resulting from stroke. This distress reduces patients’ willingness to perform health behaviors (18). It also increases the difficulty of executing these behaviors (6, 17). These effects contribute to the degradation of patients’ health behaviors.
3. Negative life events refer to various psychological stressors encountered by stroke patients during their rehabilitation. Research has found that negative life events significantly increase patients’ feelings of self-loathing and concurrently reduce their self-management capacity (35). The emotional impact of such events makes it difficult for patients to concentrate on daily self-management tasks (35).
4. Lack of benefit from disease management refers to patients’ negative expectations regarding the effectiveness of health behaviors in preventing stroke recurrence or improving prognosis. Studies have shown that patients’ beliefs about their disease prognosis are significantly associated with their health behaviors; more positive beliefs are linked to a lower likelihood of health behavior deterioration (19, 20).
5. Low self-control ability refers to a generally low level of trait-based self-control at the individual level. An individual’s self-control ability is closely related to health management. Low self-control ability is an important reason for patients’ failure to maintain health behaviors (36).
6. External misinformation refers to health information accessed by patients that contradicts scientific evidence for stroke rehabilitation. Lin et al. (37) found that a lack of stroke-related knowledge and information is a significant crisis faced by stroke survivors. This primarily manifests in two ways: unclear or insufficient information, and the inappropriate presentation of information.
7. Lack of self-regulation motivation refers to patients’ lack of intrinsic drive to proactively maintain health behaviors and avoid unhealthy ones. He et al. (38) point out that motivation is a crucial internal force for sustaining patients’ health behaviors; insufficient motivation leads to unstable and relapse-prone behavior patterns. Existing research has strengthened patient health behaviors by enhancing motivation (24).
8. Weakening of self-control refers to impulsive decision-making, reckless risk-taking, and stagnation in health behaviors resulting from the temporary weakening of cognitive, emotional, and behavioral control capacities. Its theoretical basis stems from the strength model of self-control, which posits that self-control is a finite resource subject to temporary depletion following demanding tasks (39). Extensive meta-analytic evidence indicates that such state-based ego depletion significantly increases impulsive decisions and undermines behavioral persistence (40). In the realm of health behavior, studies confirm that individuals in a state of ego depletion are more likely to make choices detrimental to long-term health (41).
9. Lack of support refers to insufficient support at the familial, social, and medical levels received by patients during rehabilitation. Research indicates that socio-familial support is also a significant influencing factor in health behavior deterioration; inadequate support acts as a barrier for patients in performing health behaviors and maintaining a healthy lifestyle (42, 43).

#### 2.4.2 Development and characteristics of the measurement instrument

The nine condition variables in this study were measured using a self-administered questionnaire. All items were derived from a prior systematic qualitative study (32) and were designed to capture the critical threshold for determining whether each specific barrier was “present.” Consequently, the questionnaire employed a dichotomous (yes/no) format. For instance, the item for “long-term high-demand tasks” was: “Do you feel that managing your post-stroke health (e.g., medication, follow-ups, exercise) is an overwhelming and demanding task?” Respondents’ answers directly formed the initial dichotomous data (yes = 1, no = 0). Specific details are provided in Table 2. Meanwhile, to ensure our dichotomous data accurately captured the clinical threshold, we implemented a standardized interview protocol: Clear instructions anchored responses to factors that actually impacted behavior over the past year. When ambiguous responses (e.g., occasionally) arose, interviewer used a standardized probe based on operational definition (“Has it frequently caused you to modify or give up planned behavior during this period?”) to guide participants to a clear clinical judgment (yes/no). This procedure ensured that the dichotomous data represented a refined assessment of behavioral impact aligned with our theoretical construct, rather than a simple recording of raw, unguided perceptions.

**Table 2:** Measurement items and psychometric properties of the conditions.

Variable (Code)	Measurement item (Yes/No)	*M* ± *SD*	Item-Total r	Cohen’s Kappa
Long-term demanding assignments (A)	Do you feel that managing your post-stroke health (e.g., medication, check-ups, exercise) is an overwhelming and demanding task?	0.51 ± 0.50	0.58	0.784
Low sense of benefit from disease management (B)	Do you believe that lifestyle changes are not useful in preventing another stroke?	0.29 ± 0.45	0.56	0.785
symptomatic distress (C)	Are you persistently troubled by noticeable post-stroke symptoms (e.g., pain, fatigue, low mood)?	0.57 ± 0.49	0.45	0.768
negative life event (D)	In the past 6 months, have you experienced any major life events that caused you significant distress?	0.06 ± 0.23	0.23	0.715
Low self-control (E)	In general, would you describe yourself as someone who easily gives up on long-term goals or has difficulty resisting temptation?	0.33 ± 0.47	0.58	0.842
Insufficient motivation for self-regulation (F)	Do you feel a lack of motivation to maintain healthy behaviors (e.g., exercise, diet)?	0.33 ± 0.47	0.72	0.677
Lack of support (G)	Do you feel a lack of sufficient understanding and support from family or friends during your recovery?	0.11 ± 0.32	0.22	0.720
External misinformation (H)	Do you often encounter or believe health information that contradicts your doctor’s advice?	0.10 ± 0.31	0.23	0.653
Weakening of self-control (I)	Recently, have you found it particularly hard to focus on completing health behaviors and more likely to act on impulse?	0.17 ± 0.37	0.50	0.785

For the sake of conciseness, variable codes (A-I) are used to report all analytical results in the following sections. Their corresponding definitions are provided in this table.

#### 2.4.3 Assessment of validity and reliability

To ensure the rigor of the measurement instrument, the following assessments were conducted:

Content Validity: All items were reviewed and revised by a panel comprising clinical neurologists, rehabilitation specialists, and health psychologists to ensure they accurately reflected the target constructs.

Construct Validity and Internal Consistency: To examine the internal consistency and structural validity of the instrument, an item analysis was performed on the nine dichotomous items. First, a total score (i.e., the sum of affirmative responses) was calculated for all items. Subsequently, the point-biserial correlation between each item and this total score was computed as an index of item discrimination. As shown in Table 2, the Kuder–Richardson Formula 20 (KR-20) reliability coefficient for the nine items was 0.57. It is important to emphasize that the variables in this study were selected as theory-driven conditions for Qualitative Comparative Analysis (QCA) (44). Their purpose was to comprehensively capture the heterogeneous mechanisms leading to health behavior deterioration, not to form a psychometric scale measuring a single latent variable. Within this context, a moderate level of internal consistency is acceptable. More theoretically informative is the pattern of item-total correlations: items representing core intra-personal barriers (e.g., lack of self-regulation motivation, long-term high-demand tasks, low self-control ability) showed higher correlations with the total score (0.58–0.72), while items representing external or situational factors (e.g., negative life events, lack of support, external misinformation) showed lower correlations (0.22–0.23). This pattern of associations meets the QCA ideal for condition variables to be “neither fully independent nor highly collinear” (29).

Test–Retest Reliability: 2 weeks after the initial assessment, a retest was administered to a subsample (*n* = 30). The Cohen’s kappa coefficients for each variable ranged from 0.65 to 0.84 (Table 2), indicating good temporal stability of the measurements.

Sensitivity Analysis of Calibration: To ensure robustness against potential variations in measurement, a sensitivity analysis was conducted. Given that the dichotomous calibration was based on patients’ self-reported yes/no responses, we employed a random perturbation analysis to simulate minor measurement errors that might arise from different interpretive thresholds. Specifically, a 5% misclassification error (i.e., randomly flipping the values of 5% of cases) was introduced into each of the nine condition variables. This process was repeated 100 times to generate perturbed datasets. Using the original analysis parameters (consistency ≥ 0.8, frequency ≥ 2), the fsQCA was re-run on each perturbed dataset. The results showed that the configuration of the core solution remained stable, appearing in over 90% of the perturbed samples. This indicates that the main findings are highly robust to minor, random fluctuations in data assignment.

#### 2.4.4 Definition and calibration of the outcome variable

The outcome variable in this study was “health behavior deterioration.” Its calibration was based on longitudinal tracking data. Latent growth mixture modeling was applied to health behavior scores at four time points over a 12-month period post-discharge for the 210 patients. The optimal model identified two behavioral trajectories: a “maintenance/improvement group” and a “deterioration group.” Patients classified into the “deterioration group” were calibrated as having the outcome occur (membership = 1); conversely, those not in this group were calibrated as the outcome not occurring (membership = 0). This method ensured that the classification of the outcome variable was based on individuals’ behavioral change patterns over time, providing an objective statistical foundation.

## 3 Results

### 3.1 Univariate necessity analysis

We first examined whether any single condition was necessary for health behavior degradation, using a consistency threshold of 0.9 (29). The results (Table 3) showed that no single condition met this criterion. Although the absence of a negative life event had a consistency above 0.9, its low coverage (<0.5) and failure to pass the relevance test indicated it was not a necessary condition (29). This key finding confirms that health behavior degradation is not driven by any one factor but requires the combination of multiple conditions.

**Table 3:** Necessity analysis for health behavior degradation.

Conditional variable	Consistency	Site coverage	Necessary condition
Long-term demanding assignments	0.797619	0.626168	No
No long-term demanding assignments	0.202381	0.165049	No
Low sense of benefit from disease management	0.452381	0.622951	No
High sense of benefit from disease management	0.547619	0.308725	No
symptomatic distress	0.654762	0.458333	No
asymptomatic distress	0.345238	0.322222	No
negative life event	0.095238	0.666667	No
Non-negative life events	0.904762	0.383838	No
Low self-control	0.535714	0.652174	No
High self-control	0.464286	0.276596	No
Insufficient motivation for self-regulation	0.690476	0.828571	No
sufficient motivation for self-regulation	0.309524	0.185714	No
Lack of support	0.142857	0.5	No
Adequate support	0.857143	0.387097	No
External misinformation	0.154762	0.590909	No
No external misinformation	0.845238	0.377660	No
Weakening of self-control	0.321429	0.771429	No
No Weakening of self-control	0.678571	0.325714	No

Necessity analysis was conducted to identify any single condition that was always present when the outcome occurred. A condition was considered potentially necessary if its consistency score was ≥ 0.90. However, to be deemed a necessary condition in a substantive sense, the condition also required a non-negligible coverage value.

### 3.2 Path configuration and analysis

Following the calibration of variables, we conducted a sufficiency analysis. The analysis employed a consistency threshold of 0.80 and a frequency threshold of 2 cases to identify configurations (25). We adopted the standard form previously by Rihoux and Ragin (25) to present the results arising from QCA analysis, using standard notation where a large symbol indicates a core condition and a small symbol a contributing condition (see Table 4 note for details). Table 4 presents the seven pathways used to explain the degradation of health behavior; each column represents one possible condition grouping. Our key findings are as follows: the overall solution exhibited high consistency (0.92) with a coverage of 0.286. Individual pathway consistencies ranged from 0.83 to 1.00, as detailed in Table 4. All individual and overall consistency values surpassed the 0.80 threshold, confirming the validity of the solutions (25).

**Table 4:** Group analysis of health behavioral deterioration in stroke patients.

Trails	A				B		
	Aa1	Aa2	Ab1	Ab2	B1	B2	B3
Long-term demanding assignments	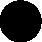	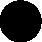	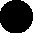	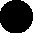	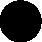	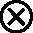	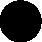
Low sense of benefit from disease management	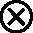	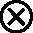		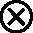	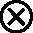	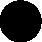	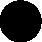
symptomatic distress	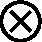	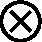	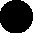		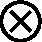	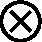	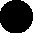
negative life event	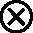	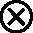	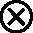	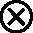	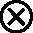	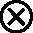	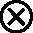
Low self-control	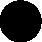	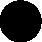	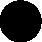	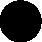	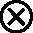	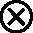	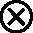
Insufficient motivation for self-regulation	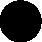	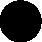	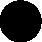	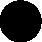	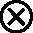	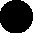	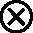
Lack of support		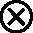	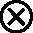	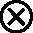	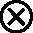	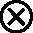	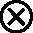
External misinformation	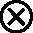	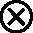	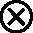	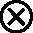	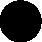	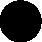	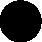
Weakening of self-control	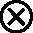		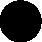	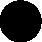	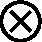	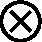	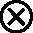
original coverage	0.083333	0.0952381	0.095238	0.0595238	0.0238095	0.0238095	0.0238095
Unique coverage	0.0238095	0	0.0714286	0	0.0238095	0.0238095	0.0238095
consistency	0.875	0.888889	0.888889	1	1	1	1
Overall consistency	0.923077						
Overall coverage	0.285714						

Black circles (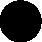) indicate the presence of a core condition; small circles (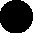) indicate the presence of a contributing (peripheral) condition. Crossed-out circles (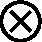) indicate the absence of a core condition; small crossed-out circles (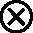) indicate the absence of a contributing condition. Blank cells indicate “do not care” situations, where the condition may be either present or absent for the configuration to remain sufficient. Pathway legend: Path labels indicate: A (endogenous type, characterized by the absence of External Misinformation) and B (exogenous type, characterized by the presence of External Misinformation). Within type A, suffix a denotes pathways without Weakening of Self-Control, while suffix b denotes pathways including this condition. Numerals (1, 2, 3) distinguish different configurations.

The 7 paths were divided into an endogenous A type that was dominated by low self-control and insufficient self-regulation motivation, and an exogenous B type that was dominated by external misinformation. Type A was further divided into Aa and Ab sub-types by another variable (a weakening of self-control or the absence of such weakening). The detailed presence/absence patterns of conditions for each pathway are summarized in Table 5.

**Table 5:** Configuration of conditions for each sufficient pathway.

Pathway type & name	Present variables (Core in Bold)	Absent variables (Core in Bold)
Endogenous: Aa1	**Long-term demanding assignments** **Low self-control** **Insufficient motivation for self-regulation**	**Symptomatic distress** **Low sense of benefit from disease management** Negative life event External misinformation **Weakening of Self-control**
Endogenous: Aa2	**Long-term demanding assignments Low self-control** **Insufficient motivation for self-regulation**	**Low sense of benefit from disease management** Symptomatic distress Negative life event Lack of support External misinformation **Weakening of self-control**
Endogenous: Ab1	**Low self-control** **Insufficient motivation for self-regulation** **Weakening of self-control** Long-term demanding assignments Symptomatic distress	Negative life event Lack of support External misinformation
Endogenous: Ab2	**Low self-control** **Insufficient motivation for self-regulation** **Weakening of self-control** Long-term demanding assignments	**Low sense of benefit from disease management** Negative life event Lack of support External misinformation
Exogenous: B1	**External misinformation** Long-term demanding assignments	**Low self-control** **Insufficient motivation for self-regulation** Low sense of benefit from disease management Symptomatic distress Negative life event Lack of support **Weakening of self-control**
Exogenous: B2	**External misinformation** Low sense of benefit from disease management Insufficient motivation for self-regulation	**Long-term demanding assignments** **Low self-control** Symptomatic distress Negative life event Lack of support **Weakening of self-control**
Exogenous: B3	**External misinformation** Long-term demanding assignments Low sense of benefit from disease management Symptomatic distress	**Low self-control** **Insufficient motivation for self-regulation** Negative life event Lack of support **Weakening of self-control**

This table lists the specific condition states for each of the seven sufficient pathways. Bold indicates a condition that is part of the core (necessary within the combination) for that pathway. Variables not listed were irrelevant (do not care) for the specific pathway. Individual and overall consistency and coverage metrics are reported in Table 4.

#### 3.2.1 Type A—endogenous

We identified two pathways for type Aa, which were characterized by the fact that in the absence of significant symptomatic distress, long-term demanding tasks caused by stroke could exacerbate the depletion of a patient’s psychological resources, especially for patients with low levels of self-control and significantly less motivated to self-regulate. Specifically, path Aa1 showed a consistency of 0.875 with a raw coverage of 0.083, while Aa2 had a consistency of 0.889 and a raw coverage of 0.095. These pathways highlight the role of long-term demanding tasks as a core stressor when self-control and motivation are low, yet self-control weakening has not yet occurred.

The Ab type was associated with two pathways, which were defined by the presence of self-control weakening as a core condition. These pathways manifested as the loss of psychological resources caused by long-term high-demand tasks, and a lack of self-control ability and self-regulation motivation. Path Ab1 (consistency: 0.889, raw coverage: 0.095) and Ab2 (consistency: 1.00, raw coverage: 0.060) both represent this high-risk psychological configuration, centered on the combination of low self-control, insufficient self-regulation motivation, and self-control weakening. In this configuration, the core conditions of this type were low levels of self-control, a lack of self-regulatory motivation, and weakened self-control. Long-term demanding tasks and symptomatic distress are the complementary conditions. Compared to the Aa sub-type, the core role in these pathways shifts from primary causes (e.g., long-term demanding tasks) to the self-control deficit configuration itself, as the regulatory system is already compromised.

#### 3.2.2 Type B—exogenous

The three exogenous pathways (B1, B2, B3) applied to a distinct patient profile characterized by relatively strong self-control and adequate self-regulation motivation. All three paths demonstrated perfect consistency (1.00). Across all three, external misinformation served as the core condition. They differ in their ancillary stressors: B1 (unique coverage: 0.024) combines external misinformation with long-term demanding tasks; B2 (unique coverage: 0.024) combines it with a weak sense of disease benefit; and B3 (unique coverage: 0.024) combines it with both demanding tasks and a weak sense of benefit, plus symptom distress. In this type, the negative effects of long-term demanding tasks or a poor sense of disease benefit combined with the emergence of erroneous external information can lead to an inability to replenish depleted psychological resources, thus reducing adherence to health behavior and leading to behavioral deterioration.

### 3.3 Robustness tests

To verify the stability of the identified configurations, we conducted a robustness check by applying stricter analytical thresholds (consistency threshold: 0.85; frequency threshold: 3). Under these criteria, the results were refined to the most robust core (complete comparison is provided in Table 6). Of the original seven pathways, only two demonstrated high robustness. Both pathways are endogenous and share the core condition combination E*F*I (where E = low self-control, F = insufficient motivation for self-regulation, I = weakening of self-control). This result establishes “the depletion of self-control (E) and regulatory motivation (F) resources, leading to the state-like weakening of self-control (I)” as the most stable and replicable core psychological mechanism for endogenous health behavior degradation. The other pathways that did not meet the new frequency threshold (including exogenous pathways and other endogenous variants) represent alternative configurations that are equally sufficient under specific circumstances. The coverage of the overall solution decreased from 0.286 to 0.107, reflecting the analysis’s focus on more universal core cases, while the consistency of the overall solution remained high at 0.900, ensuring the reliability of the robust pathways.

**Table 6:** Comparison of sufficient pathways before and after robustness test.

Solution & path	Condition combination	Original coverage	Consistency	Survives robustness test (0.85/3)?
Original Solution (0.8/2)		0.286	0.923	
Aa1	A* ~ B* ~ C* ~ D*E*F* ~ H* ~ I	0.083	0.875	No
Aa2	A* ~ B* ~ C* ~ D*E*F* ~ G* ~ H	0.095	0.889	No (closely resembles Path 2)
Ab1	A*C* ~ D*E*F* ~ G* ~ H*I	0.095	0.889	Yes (as Robust Path 2)
Ab2	A* ~ B* ~ D*E*F* ~ G* ~ H*I	0.060	1.000	No
B1	A* ~ B* ~ C* ~ D* ~ E* ~ F* ~ G*H* ~ I	0.024	1.000	No
B2	~A*B* ~ C* ~ D* ~ E*F* ~ G*H* ~ I	0.024	1.000	No
B3	A*B*C* ~ D* ~ E* ~ F* ~ G*H* ~ I	0.024	1.000	No
Robust Solution (0.85/3)		0.107	0.900	
Robust Path 1	A* ~ B* ~ C* ~ D*E*F* ~ G* ~ H*I	0.036	1.000	Yes
Robust Path 2	A*B*C* ~ D*E*F* ~ G* ~ H*I	0.071	0.857	Yes

Variable codes (A, B, C, D, E, F, G, H, I) used in this table are consistent with Table 2. In the configuration expressions, a tilde (~) preceding a variable indicates the absence of that condition (e.g., ~B denotes the absence of “Lack of benefit from disease management”), while a variable without ~ indicates its presence.

## 4 Discussion

In this study, we identified two predominant typologies in the degradation of health behaviors among stroke patients: an endogenous type, driven by deficits in self-control and self-regulation motivation, and an exogenous type, precipitated by exposure to external misinformation. Under the endogenous type, patients exhibit poorer self-control and significantly diminished self-regulatory motivation. This type comprises two sub-categories. In the first, patients have not yet developed a marked weakening of self-control and report minimal symptom distress; however, the long-term demanding tasks inherent to disease management create a substantial perceived burden, leading to a higher likelihood of health behavior degradation. In the second sub-category, patients are already experiencing a weakening of self-control, which serves as the core condition. In this state, the impact of long-term demanding tasks and symptomatic distress on behavior degradation appears attenuated. Conversely, under the exogenous type, patients generally possess strong self-control and adequate self-regulatory motivation. The primary issue lies in exposure to external misinformation, which, in combination with contextual stressors such as long-term demanding tasks and a weak sense of disease benefit, ultimately leads to health behavior decline.

Furthermore, a robustness check (applying stricter consistency and frequency thresholds) refined these findings, revealing a core condition combination within the endogenous type: E*F*I (Low self-control, Insufficient motivation for self-regulation, Weakening of self-control). This triad constitutes a robust, generalized psychological mechanism, integrating a stable trait deficit (E), a motivational driver (F), and a critical proximal state (I). This configuration aligns with and extends theoretical models of self-regulatory failure, which posit that the depletion of self-control resources is contingent upon both trait capacity and motivational factors (39). More importantly, the robustness check elucidates a typological-hierarchical structure within the observed equifinality. First, it corroborates the fundamental distinction between endogenous and exogenous pathways, defined by the presence or absence of core psychological deficits. Second, within the endogenous spectrum, it pinpoints the E*F*I combination as the most stable and central mechanism. Other endogenous pathways and the distinct exogenous pathway represent alternative, context-sufficient configurations leading to the same outcome. This refined framework—delineating primary typologies and a robust endogenous core—offers a nuanced map for understanding patient heterogeneity.

Compared with previous studies (14, 19, 20), the exploration of multiple pathways can better provide appropriate programs for improving or preventing the deterioration of health behaviors in patients. The results suggested that we should attach importance to identifying patients’ self-control ability and the authenticity of external information sources and contents. Building on our analytical framework, interventions must be correspondingly tailored. For patients on endogenous pathways, priority should be given to addressing the core psychological vulnerability (E*F*I). This involves targeted strategies for those with low self-control, including close monitoring for the emergence of state-like weakening and implementing motivational interventions to bolster self-regulation drive. Adjusting disease management demands to alleviate perceived burden is also crucial. For patients whose profile aligns with the exogenous pathway (characterized by preserved self-control), we should pay more attention to the source and content of information they acquire, correct misinformation, challenge misconceptions in a timely manner, strengthen disease education for the patient’s families and friends, and provide convenient and correct information channels to counteract the influence of misleading content.

The long-term high-demand task was consistently present as a pivotal contextual stressor across pathways, confirming its role as a key driver of perceived burden. The strict secondary prevention measures for stroke lead to an increased sense of disease burden for the patient and can drive the degradation of their health behaviors. These findings regarding the burden of long-term demanding tasks were consistent with those reported by previous studies (45, 46). A heavier self-perceived burden will induce a negative emotional experience in patients. Thus, patients’ self-esteem and treatment confidence decline, adversely impacting healthy behaviors, such as exercise and a healthy diet (45). In addition, the heavy burden of self-perception will also cause patients to consider economic problems in more detail, especially the cost of long-term medication and examination, thus influencing patients’ confidence in their recovery and reducing health behaviors (46). However, despite the repeated occurrence of self-perception as a contributing condition, condition analysis showed that this was not a necessary condition for the deterioration of health behaviors. Therefore, we should adopt a holistic, configurational perspective, focusing on the identification and interaction of multiple condition variables rather than single factors, to formulate intervention programs.

Methodologically, analysis in this study revealed the configurational nature underlying health behavior degradation, demonstrating that no single factor constitutes a necessary condition; rather, it is the conjunction of multiple conditions that forms sufficient pathways. The robustness test further distilled a core endogenous psychological mechanism—the combination of low self-control, insufficient motivation for self-regulation, and weakening of self-control—providing a key target for prioritized clinical intervention. This finding transcends the traditional single-factor perspective and lays the groundwork for developing precise strategies tailored to complex etiologies.

Building on this, immersive technologies such as Virtual Reality (VR) present a highly promising avenue for achieving such precision intervention (47). VR can transform demanding, long-term tasks into gamified experiences to reduce perceived burden, enhance self-regulation motivation and self-control capabilities through immediate feedback and structured practice (48), and provide patients with accurate, standardized guidance within an immersive environment. Preliminary evidence supports its safety and feasibility in stroke rehabilitation (49). Crucially, the success of such technologies hinges on patient-centered design principles to ensure the intervention’s appeal and personal relevance (50). Therefore, the core contribution of this study lies in translating the complex causes of behavioral decline into clear, configurational pathways that are amenable to targeted intervention. This provides clear guidance for future research to validate technology-driven, personalized rehabilitation programs.

## 5 Limitations and future studies

This study has several limitations that warrant consideration and offer directions for future research. First, the measurement of condition variables relied primarily on patient self-report. Although validated scales and sensitivity analyses were employed, integrating objective behavioral metrics (e.g., from wearable devices) in future studies would strengthen validity and causal inference. Second, the aggregation of multiple health behaviors into a composite outcome variable, while clinically meaningful for exploring generalized deterioration, may obscure the unique mechanisms underlying specific behaviors like medication adherence or exercise. Future studies with larger samples could analyze these behaviors separately. Third, the selection of condition variables, though theory-driven, involves inherent subjectivity, and other relevant factors may exist. Fourth, the sample was drawn from a single center in Suzhou, which may affect the generalizability of the identified pathways. Expanding the geographical and institutional scope is recommended.

## 6 Conclusion

We tracked the health behavior of stroke patients over the first year post-discharge and investigated the pathways associated with health behavior degradation based on nine variables. Our analysis identified two primary archetypes of pathways: an endogenous type, predominated by deficits in self-control and self-regulation motivation, and an exogenous type, driven primarily by exposure to misinformation. Moreover, robustness tests confirmed a stable core configuration (low self-control * insufficient motivation * self-control weakening) within the endogenous archetype, underscoring its central role. Health behavior degradation does not stem from single factors but from specific configurations of multiple concurrent conditions. Consequently, moving beyond isolated-factor approaches to adopt a holistic, configurational perspective is essential for assessment and intervention. Practically, this implies interventions should be dual-pronged: they must simultaneously target the stable psychological core (e.g., through self-control reinforcement) and address diverse contextual risk combinations (e.g., by ensuring support and correcting misinformation). Future research should leverage tools like VR/AR for personalized intervention and incorporate objective measures to validate these pathways.

## Data Availability

The original contributions presented in the study are included in the article/supplementary material, further inquiries can be directed to the corresponding authors.
